# Dynamics of the canine gut microbiota of a military dog birth cohort

**DOI:** 10.3389/fmicb.2025.1481567

**Published:** 2025-03-24

**Authors:** Jae-Uk An, Seung-Hyun Mun, Woo-Hyun Kim, Je Kyung Seong, Kyoungwan Park, Seongbeom Cho

**Affiliations:** ^1^College of Veterinary Medicine and Research Institute for Veterinary Science, Seoul National University, Seoul, Republic of Korea; ^2^Korea Mouse Phenotyping Center (KMPC), Seoul National University, Seoul, Republic of Korea; ^3^Military Working Dogs Training Center, Gangwon, Republic of Korea; ^4^Comparative Medicine Disease Research Center (CDRC), Seoul National University, Seoul, Republic of Korea

**Keywords:** military dogs, birth cohort, gut microbiota, growth, controlled environment, 16s rRNA amplicon sequencing, *Enterococcus faecalis*, Canine parvovirus-2

## Abstract

**Introduction:**

We systematically tracked early life stages in a military dog birth cohort to investigate canine gut microbiota dynamics related to environmental exposure during growth. This study utilized 16s rRNA amplicon sequencing-based analysis with molecular epidemiology of *Enterococcus faecalis* within a controlled environment at a military dog training center.

**Methods:**

We examined shifts in gut microbiota diversity and taxonomic composition across four growth stages (lactation, weaning, starter, puppy) in three littermate groups. Additionally, *E. faecalis* dynamics was analyzed to confirm strain sharing among littermate groups.

**Results:**

Gut microbiota changed rapidly during early growth, stabilizing at the puppy stage. This is supported by increased similarity in taxonomic composition among littermate groups, as they experienced an increased shared external environment and consumed the identical diets. *E. faecalis* strain sharing among littermate groups increased as dogs aged. Nine *E. faecalis* cluster types were identified; three specific types (type I, II, and IX) dominated in each littermate group during lactation. With greater exposure to the shared external environment, cluster type I gradually assumed dominance across all groups. Despite the dynamic shifts in microbiota, we found five genera within the core microbiota, *Bacteroides*, *Peptoclostridium*, *Fusobacterium*, *Lactobacillus*, and *Blautia*.

**Discussion:**

This study is the first to explore the dynamic nature of early-life canine gut microbiota, illustrating its transition to stability and its resilience to environmental perturbations within the controlled training environment of a military dog birth cohort.

## 1 Introduction

Gut microbiota includes all the microorganisms in the gastrointestinal (GI) tract, including bacteria, viruses, fungi, archaea, and protozoa (Honneffer et al., 2014). It forms a complex symbiotic relationship with the host, facilitating nutrient digestion and absorption and/or preventing the entry of exogenous pathogens (Hooda et al., 2012). However, this symbiotic relationship can be altered and reconstructed by numerous factors, including dietary or living environmental changes, diseases, antibiotic use, genetics, and aging (Cho and Blaser, 2012; O’Toole and Jeffery, 2015; Million et al., 2017). Among those factors, strong perturbations, including infectious disease and antimicrobial application, can cause dysbiosis, a disrupted symbiotic state (Sommer et al., 2017).

In clinical studies, environmental factors during early life stages play a critical role in gut microbiota establishment and development (Kelsen and Wu, 2012). A well-established gut microbiota at the early life stages may assist the host in developing resilience to microbial dysbiosis and diseases (Lozupone et al., 2012; Parkin et al., 2021). Gut microbiota management can be a powerful tool for preventing and treating various diseases (Markandey et al., 2021; Waller et al., 2021). The dysbiosis patterns in humans and dogs are relatively similar (Coelho et al., 2018). Understanding the development of healthy canine gut microbiota in early life stages can provide a valuable cornerstone for health care and disease treatment in adult dogs.

Microbiota is greatly affected by external factors; thus, controlling for confounding factors is important when studying microbiota changes (Karahan, 2024). Domestic dogs interact with various external factors in indoor and outdoor environments (Pocar et al., 2023). Therefore, excluding confounding factors is difficult. The health state of military dogs used for special purposes is crucial, and they live systematically under strict veterinarian management. They are born and raised in an controlled training environment (Military Dog Training Center), and variations in individual dietary habits and living conditions are minimal. The living environment characteristics of these military dogs make them suitable models for studying canine gut microbiota.

*Enterococci*, particularly *E. faecalis* and *E. faecium*, are crucial components of the gut microbiota in humans and animals and have been suggested to be early gut colonizers (Fisher and Phillips, 2009; Gilmore et al., 2013). Colonization with *E. faecalis* may help maintain gut microbiota homeostasis by reducing the risk of infectious diseases and stimulating immune responses in early life stages (Wang et al., 2008). In many instances, sequencing-based metagenomic analysis, such as 16s rRNA analysis or shotgun metagenomics, cannot accurately differentiate between closely related microbial strains owing to technical limitations, such as sequencing errors (Yan et al., 2020). In contrast, culture-based molecular methods allow distinguishing between different species with similar phenotypic characteristics and between strains belonging to the same species (Martin-Platero et al., 2008). In addition to 16S rRNA metagenomic analysis, analyzing genetic relationships using culture-based methods can be an important key factor in understanding the evolution and dynamics of microbial communities at the strain level (Leventhal et al., 2018; Lou et al., 2021; Goyal et al., 2022; Wolff et al., 2023).

This study aimed to track the dynamics of the gut microbiota of dogs at early life stages (lactation/weaning/starter/puppy) by using a birth cohort group in a military dog training center. We analyzed the gut microbiota of military dogs that were born and raised in a training center without contact with external environments. The α- and β-diversities, taxonomic composition, and predictive functional profile were analyzed by growth stage based on 16s rRNA amplicon sequencing-based analysis to determine gut microbiota changes. Additionally, the strain-level dynamics of *E. faecalis*, a representative fecal indicator bacterium, were analyzed using rep-PCR to confirm the sharing, proliferation, and introduction of bacterial strains among littermates.

## 2 Materials and methods

### 2.1 Fecal sample collection from military dogs

Fecal samples were collected from 27 dogs born and raised at the Military Working Dog Training Center located in Gangwon-do, Republic of Korea, between May 2016 and March 2017. Since the puppies were delivered from three different mothers, they were classified into three groups (A, B, and C), each comprising puppies from the same mother. There were 7, 10, and 10 dogs in groups A, B, and C, respectively. The breed of the dogs in group A was German Shepherd, and that of groups B and C was Belgian Malinois. To collect the fecal samples, a clean and independent space was provided for each dog in the morning after feeding. If a fresh fecal sample could not be collected at any time point for a particular individual, all samples from that individual across the seven time points were excluded from the analysis. Consequently, the final numbers of animals included in the analysis were six, eight, and six animals in groups A, B, and C, respectively. The fecal samples were collected in sterilized containers immediately after excretion and placed into Styrofoam packages containing a sufficient amount of ice packs to maintain ice-cold conditions (∼4°C) during transportation to the laboratory.

**FIGURE 1:**
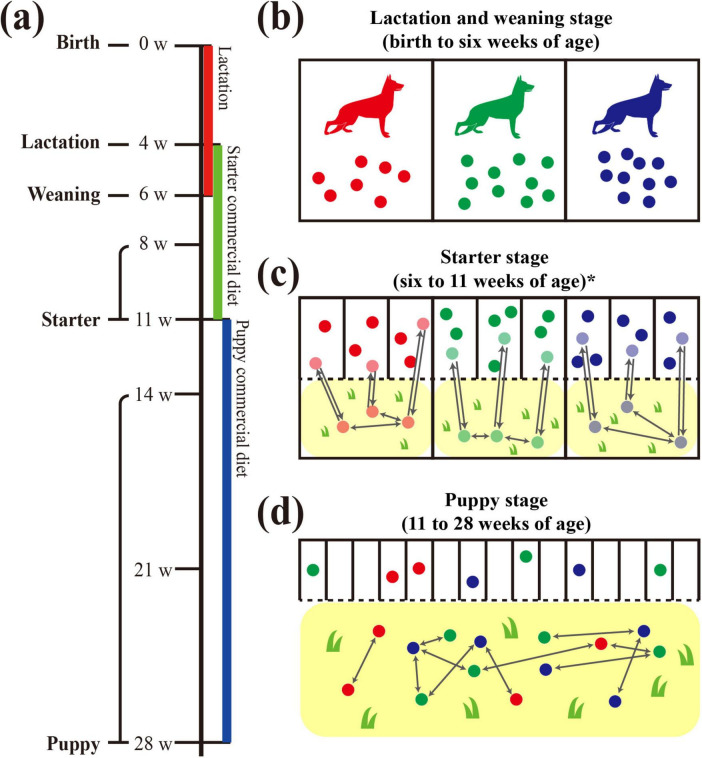
Graphical diagram of dog growth and living environment in the military dog training center. Schematic diagram for changes in the living environment from birth to 28 weeks of age of three littermate groups. **(a)** Sampling was performed at 4, 6, 8, 11, 14, 21, and 28 weeks of age, and the dietary stages were divided into lactation (4 weeks of age), weaning (6 weeks of age), starter (8 and 11 weeks of age), and puppy (14, 21, and 28 weeks of age). Colored bars below the timeline indicate the provided diet: lactation diet in red, starter commercial diet in green, and puppy commercial diet in blue. **(b)** Separate living spaces (indoors, cement, metal fence) were provided for each littermate group with the mother dog from birth to the weaning stage (6 weeks of age). **(c)** Three to four littermates shared one indoor living space (cement, metal fence), and separate backyards (outdoors, soil and grass) were also provided for each littermate group. **(d)** An individual living space and shared outdoor training field were provided for each puppy. In the training field, contact occurred freely between puppies, regardless of littermate group.

Sampling was performed at seven points between 4 and 28 weeks of age, and the sampling points were grouped into four growth stages according to growth and dietary changes (Figure 1): lactation (4 weeks of age), weaning (6 weeks of age), starter (8 and 11 weeks of age), and puppy (14, 21, and 28 weeks of age). The feed differed depending on the growth stage: mother’s breast milk only during the lactation period; mother’s breast milk with a starter commercial diet (Royal Canin, Aimargues, France) during the weaning period; starter and puppy commercial diets only (Royal Canin) during the starter and puppy stages, respectively. The diet and its nutrients composition provided to military dogs in our study are explained in order to provide complete information to the reader. The nutritional composition of the commercial diets was crude protein (min) 28.0%, crude fat (min) 20.0%, crude fiber (max) 3.4%, and moisture (max) 10.0% for the starter commercial diet; and crude protein (min) 32.0%, crude fat (min) 12.0%, crude fiber (max) 3.4%, and moisture (max) 10.0% for the puppy commercial diet. The feed and or its ingredients was not changed/manipulated for this study, compared to the age-specific feed originally provided at Military working dogs Training center.

According to the growth stages, military dogs were provided with different living spaces. During the lactation and weaning stages, the siblings lived together, sharing the living space with their mother (Figure 1). During the starter stage, sibling dogs were housed separately from their mother in individual living spaces, with three to four dogs per space. Although their sleeping and feeding areas were separated, these spaces were connected to a shared fenced backyard playground, allowing all siblings to interact with one another. However, this backyard was not shared with other sibling groups. From the puppy stage, each individual dog lived independently in a separate space, using the same building regardless of the sibling group. Furthermore, they started to perform outdoor field training from this stage (14 weeks of age) to develop qualities suitable to military dogs. This age was a period of adaptation to field training, which actively began from the age of 21 weeks.

Among groups A, B, and C, only the puppies in group B exhibited diarrhea suspected to be infectious bowel disease around 13 weeks of age, while those in groups A and C were healthy and without any disease symptoms. At 13 weeks and 6 days of age, Canine parvovirus-2 (CPV-2) infection was diagnosed in group B using the “Antigen Rapid CPV Ag Test Kit” (BIONOTE Inc., Gyeonggi-do, Korea). Accordingly, group B received antibiotics (enrofloxacin, cefazolin, amoxicillin/clavulanic acid, and metronidazole) and intravenous fluid therapy for approximately two weeks until the symptoms vanished.

### 2.2 Study design

Figure 1 shows an overview of the sampling timeline. To analyze healthy canine gut microbiota according to age, we included fecal samples from 4, 6, 8, 11, 14, 21, and 28 weeks-old dogs in groups A and C, and 4, 6, 8, and 11 weeks-old dogs in group B. The CPV-2 infection at 13 weeks in group B along with antibiotic treatment for 2 weeks could be a confounding factor; hence, we excluded the samples older than 11 weeks of age (14, 21, and 28 weeks of age) in group B.

To analyze the recovering potential of gut microbiota against CPV-2 infection during the early life stages of military dogs, we compared the gut microbiota of eight dogs in group B (CPV-infected at 13 weeks) with that of six dogs in group C (healthy, without symptoms; control group, CON) from 8 to 28 weeks of age. For clarity of the analysis, we designated group B as “group CPV” and group C as “group CON.” Further, group CPV was subdivided into CPV-high and -low based on the infection dose of CPV-2; the five dogs with a higher cycle threshold (Ct) value of the VP2 gene of CPV-2 relative to the bacterial 16s rRNA in the gut microbiota were assigned to the “CPV-high” (mean ΔCt = 1.670; 95% CI, 1.409–1.931) and the three dogs with a lower Ct value relative to 16s rRNA in the gut microbiota were assigned to the “CPV-low” group (mean ΔCt = 5.504; 95% CI, −6.245, −4.763; Supplementary Figure 1). Four of the five dogs in the group CPV-high died between weeks 11 and 14, and the fifth dog died between weeks 21 and 28. All three dogs in group CPV-low survived after 28 weeks of age.

### 2.3 Isolation and rep-PCR analysis of *Enterococcus faecalis* for culture-dependent genetic diversity analysis

Repetitive sequence-based PCR (rep-PCR) was performed to investigate whether the genetic diversity among *E. faecalis* belonging to the gut microbiota of each individual is similar to the taxonomic composition of the gut microbiota between individuals.

To isolate *Enterococcus* sp., 1 g of fecal sample was homogenized in 9 mL of Enterococcosel™ broth (Becton Dickinson and Company, United States) and incubated at 37° for 18 h. One loop of enriched broth was streaked on an Enterococcosel™ agar plate (Becton Dickinson and Company, United States) and incubated at 37°C for 18–20 h, and six suspected colonies were picked for each plate. Genomic DNA was extracted from each colony using the Instagene™ matrix (Bio-Rad, United States), and *E. faecalis* was identified using the *Enterococcus* genus-specific primer set and *E. faecalis* species-specific primer set as previously reported (Kariyama et al., 2000).

In total, 81 *E. faecalis* strains were isolated, with each strain obtained from the fecal sample of a unique individual dog within the healthy group. The rep-PCR was performed using BOXA1R primer (5′-CTA CGG CAA GGC GAC GCT GAC G-3′) and PCR mixture as follows, 12.5 μL of 2X Lamp *taq* master mix (Biofact, Korea), 2 μL of primer (25 pmol/μL), 5 μL of DNA template, and distilled water to attain a final volume of 25 μL. PCR reactions were performed using the conditions previously described (Koeuth et al., 1995). The extracted DNA quality was assessed using a Nanodrop spectrophotometer (Thermo Fisher Scientific, United States), and samples having a 260/230 absorbance ratio value above 1.8 were used for further analysis. PCR products were electrophoresed on a 1.0% agarose gel containing DNA staining solution and imaged using Gel-doc (Bio-Rad, United States). Band patterns from rep-PCR were analyzed using BioNumerics version 6.6 (Applied Maths, Belgium). Dendrograms were generated based on the Dice similarity coefficient with the unweighted pair group method with arithmetic mean using parameters as follows: 3% of optimization and 1.5% of band matching tolerance. Effective number of species (ENS) of Shannon (*e^H^*) and Simpson (1/D) (Simpson, 1949) diversity indices were used to calculate the genetic diversity of *E. faecalis* isolated at each week of age based on 80% band pattern similarity criteria. The 80% band pattern similarity criteria were applied based on previously established thresholds for rep-PCR studies (Versalovic et al., 1991; Koeuth et al., 1995).

### 2.4 Metagenomic DNA extraction and bacterial 16s rRNA amplicon sequencing

Immediately after being transported to the laboratory, the fecal samples were homogenized with a 10-fold dilution in 0.85% NaCl solution. A 250 μL aliquot of each diluted sample was used for metagenomic DNA extraction using the FastDNA™ SPIN Kit for Soil (MP Biomedicals, Irvine, CA, United States) and FastPrep^®^-24 Classic Instrument (MP Biomedicals). The DNA concentration and purity were assessed using PicoGreen (Invitrogen, Carlsbad, CA, United States) and a NanoDrop spectrophotometer (Thermo Fisher Scientific, Waltham, MA, United States). The V3–V4 region of the 16S rRNA gene was amplified using bacterial primers (Forward: 341F, 5′-CCT ACG GGN GGC WGC AG-3′; Reverse: 805R, 5′-GAC TAC HVG GGT ATC TAA TCC-3′) as described by Klindworth et al. (2013). A limited-cycle PCR was subsequently performed to add multiplexing indices and Illumina-compatible sequencing adapters for library preparation. The 16S rRNA gene amplicons were pooled and normalized using PicoGreen, and the library size was validated using the LabChip GX HT DNA High Sensitivity Kit (PerkinElmer, Waltham, MA, United States). Sequencing was conducted using the MiSeq™ platform (Illumina, San Diego, CA, United States) with a paired-end read length of 2 × 300 bp, provided by Macrogen (Seoul, Korea).

Raw sequence data were subjected to quality control to ensure clean and reliable reads for downstream analysis. Adapter sequences were removed using Scythe (v0.994) and low-quality bases were trimmed using Sickle. Reads shorter than 38 bp after adapter trimming were discarded to produce high-quality and clean data for further analysis.

### 2.5 Taxonomic assignment using 16s rRNA amplicon sequencing

Bioinformatics analysis was performed using QIIME2 (2023.9) (Bolyen et al., 2019). The raw sequence reads were processed using the Divisive Amplicon Denoising Algorithm 2 (DADA2) plugin (Callahan et al., 2016) within QIIME2, ensuring high-quality and reliable data for downstream analysis. In the denoising process, primer sequences were automatically removed from the raw reads, and low-quality bases were filtered based on a Phred quality score threshold (≥ Q20). Reads with ambiguous bases or consistently low-quality regions were discarded, while the remaining reads were truncated at the position where the average quality dropped below the threshold to minimize sequencing errors. Additionally, identical reads were dereplicated to reduce redundancy and computational load, and chimeric sequences were identified and removed *de novo* to eliminate amplification artifacts. Low-abundance amplicon sequence variants (ASVs) (< 0.25% relative abundance per sample) were removed to exclude spurious ASVs. The alignment of ASVs was accomplished using MAFFT (Katoh et al., 2002) and phylogenetic tree was analyzed with the FastTree2 plugin (Price et al., 2010). Taxonomic assignment was performed using the SILVA database (release 138) (Quast et al., 2013). After taxonomic assignment, archaeal, eukaryotic, mitochondria, and chloroplasts ASVs were filtered out to collect bacterial ASVs.

After taxa and abundance filtering, 7,031,749 sequence reads were obtained, ranging from a minimum of 19,936 to a maximum of 120,662 (1st quartile: 43,681.25, Median: 51,883.5, 3rd quartile: 64,507.25) from the 130 samples (42, 46, and 42 samples from each group). Taxonomic annotation identified a total of 9 phyla, 13 classes, 31 orders, 52 families, and 115 genera. During taxonomic assignment, sequences that could not be confidently classified at the genus or species levels were handled based on their taxonomic resolution at higher ranks. Among the seven unassigned genera observed in the dataset, two genera (g__uncultured; s__uncultured_bacterium and g__uncultured; s__uncultured_Allobaculum) within the family Erysipelotrichaceae were collapsed into a single genus, uncultured_Erysipelotrichaceae, due to their shared family-level classification. In contrast, the remaining five unassigned genera were associated with unique families; thus, they were retained as distinct taxa to preserve the taxonomic diversity within the dataset.

### 2.6 Taxonomic diversity analysis based on 16s rRNA amplicon sequencing: α- and β-diversity analyses

To further address differences in sequencing depth across samples, rarefaction was performed at a depth of 19,936 reads, which corresponds to the lowest sequencing depth among all samples. Using these normalized and rarefied datasets, four α-diversity indicators were calculated, namely Shannon diversity (ENS), Richness (observed features), Pielou’s evenness, and Faith’s phylogenetic diversity. In addition, to identify the difference in taxonomic composition between growth stages and the variance within stages, a β-diversity analysis based on the generalized UniFrac distance (gUniFrac, α = 0.5) was performed (Chen et al., 2012). β-diversity allows the comparison and verification of compositional differences in gut microbiota between individuals, and non-metric multidimensional scaling (NMDS) indirectly provides a value for compositional differences within groups. Large difference in the taxonomic composition of the gut microbiota between individuals within the same group is reflected in a wide dispersion of their coordinates on the NMDS plot. To quantify within-group dispersion, we calculated the mean and standard deviation of all pairwise gUniFrac distances within groups, and between-group significance was assessed using a *t*-test. All α- and β-diversity indices were calculated using the QIIME2 and vegan packages in R and visualized on the NMDS plot using the “Vegan” and “ggplot2” packages in R (Oksanen et al., 2007; Bolyen et al., 2019).

### 2.7 Predictive functional profiling based on taxon

To determine the predicted metabolic pathways corresponding to individual microbiota, a phylogenetic investigation of communities by reconstruction of unobserved states 2 (PICRUSt2) was performed based on the MetaCyc database (Krieger et al., 2004; Douglas et al., 2020). To assess significant differences in predicted functional pathway abundances between growth stages (Lactation-Weaning vs. Starter-Puppy stages), the Welch’s *t*-test adjusted with the Benjamini-Hochberg false discovery rate (FDR) method was used (differences in mean proportion > 0.1, *q*-value < 0.05).

### 2.8 Relative quantification of CPV-2

To quantify the infectious dose of CPV-2 in the metagenomic DNA of the eight dogs in groups CPV-low and CPV-high, we conducted quantitative real-time PCR (qRT-PCR) using the VP2 gene of CPV-2 as the gene of interest and the bacterial 16s rRNA as the endogenous control (Kumar and Nandi, 2010; Bacchetti De Gregoris et al., 2011). The VP2 gene was detected from the metagenomic DNA of the eight dogs in groups CPV-low and CPV-high at 14 weeks of age but not detected at 8, 11, 21, and 28 weeks of age. The VP2 gene was not detected from the metagenomic DNA of the six dogs in group CON at all ages.

Serial 2-fold dilutions of metagenomic DNA at concentrations of 1 × 2^0^ ng/μL to 1 × 2−^4^ ng/μL were prepared in TE buffer to generate a standard curve of 16s rRNA and CPV-2. The assay showed linearity over the dilution range with R^2^ values of 0.984 and 0.990 and reaction efficiencies of 103.3% and 108.6%, respectively. The qRT-PCR was performed using the PowerSYBR^®^ Green PCR Master Mix (Applied Biosystems, Waltham, MA, United States) and the QuantStudio™ three Real-Time PCR System thermal cycler operated by QuantStudio™ Design and Analysis desktop software v1.5.0 (Thermo Fisher Scientific, Waltham, MA, United States). Each qRT-PCR was performed in a total volume of 50 μL, containing 1 μL template DNA, 1 pmol of each primer, 25 μL 2X qPCR master mix, and 23 μL nuclease-free water. The qRT-PCR conditions consisted of initial denaturation at 95°C for 10 min, 40 cycles of 95°C for 30 s, 55°C for 30 s, and 72°C for 30 s, followed by melting curve analysis. Amplification and denaturation data were acquired for further analysis. All experiments were performed in triplicate. The relative quantity of CPV-2 was calculated using the value of ΔCt as follows: Δ*Ct* = *Ct* (Ge*ne ofinterest* : *CPV*-2) − *Ct*(Endogenouscontrol : 16s rRNA).

### 2.9 Statistical analyses

To investigate the association between alpha-diversity of gut microbiota and canine growth stages, we utilized linear mixed-effect model in R version 4.4.0. The dependent variable in our model was four alpha diversity indices. The independent variable was a categorical variable “weeks of age” and “growth stage,” respectively. To control for potential confounding factors, we used the littermate group and sex as fixed effects, and the individual puppy as a random effect to account for repeated measures within the same puppy. For post-hoc pairwise multiple comparison, the *p*-value was corrected by Bonferroni method.

To analyze the difference in taxonomic composition across growth stages, pairwise permutational multivariate ANOVA (PERMANOVA) with 999 random permutations using adonis function was performed. We performed Beta Dispersion analysis using the betadisper function in R to validate the homogeneity of multivariate dispersion across groups. The results indicated significant differences in dispersion (ANOVA, *p* < 0.001), suggesting caution in interpreting PERMANOVA results. The littermate group and sex were included in the formula of adonis as covariates. For *post hoc* pairwise multiple comparison, the *p*-value was corrected using the Bonferroni method.

To robustly investigate the significant differences in the relative taxa abundance among growth group, we used two statistical tools—generalized linear models implemented in multivariate association with linear models (MaAsLin2) and analysis of composition of microbiomes with bias correction 2 (ANCOM-BC2). Zero imputation was not performed because both differential abundance analysis tools have built-in mechanisms to handle zero values without additional preprocessing. The dependent variable in our model was relative abundance of family-level taxa for MaAsLin2 and absolute abundance of family-level taxa for ANCOM-BC2. The independent variable was a categorical variable growth stage, with the lactation stage as a reference. To control for potential confounding factors, we used the stage and sex as fixed effects, and the littermate group as a random effect to account for repeated measures. For MaAsLin2, we used LOG transformation, minimum abundance of 0.001%, individual prevalence of 10%. For ANCOM-BC2, we used minimum abundance of 100 read counts, individual prevalence of 10%, and structure zero “TRUE” option. In addition, log-fold changes were obtained from a log-linear model and *p*-values were calculated using a two-sided Z-test for ANCOM-BC2 analysis. The *q*-value was calculated using the Benjamini-Hochberg method for multiple testing correction in both the MaAsLin2 and ANCOM-BC2 analyses.

We used the Kruskal-Wallis test for alpha diversity of microbial taxa between CPV-high, CPV-low, and CON group across age of weeks 8, 11, 14, 21, and 28. To investigate the significant differences in the relative taxa abundance associated with CPV-infection, we used generalized linear models implemented in MaAsLin2, adjusting for covariate sex. The dependent variable in our model was relative abundance of genus-level taxa. The independent variable was three CPV groups including CPV-high, CPV-low and CON groups. For MaAsLin2, we used LOG transformation, minimum abundance of 0.001%, individual prevalence of 10%. The *q*-value was calculated using the Benjamini-Hochberg method for multiple testing correction.

## 3 Results

### 3.1 Growth stage-associated changes in gut microbiota α-diversity in healthy military dogs

**FIGURE 2:**
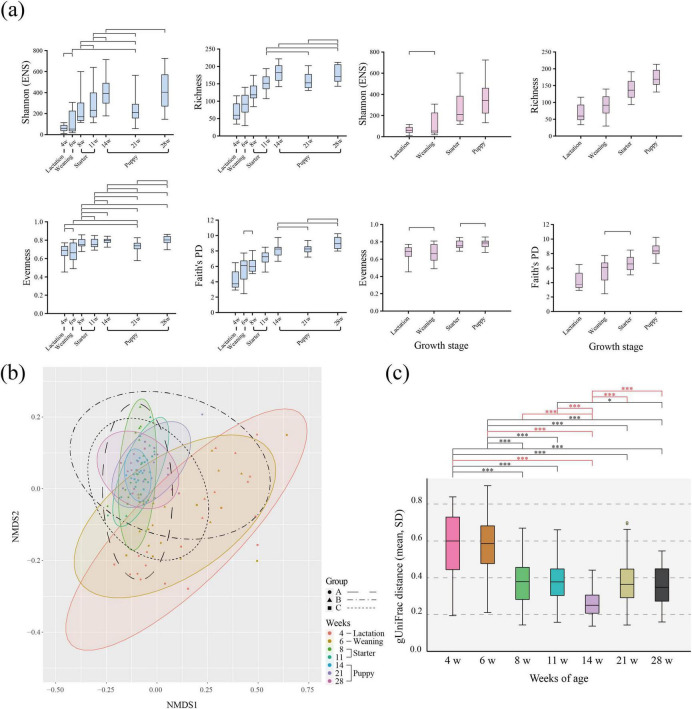
α- and β-diversity of gut microbiota according to the age (in weeks) of healthy 116 individual military dogs. **(a)** Box-and-whiskers plots represent the inter-quartile range and minimum to maximum values of α-diversity [Shannon (ENS), Richness, Pielou’s evenness, Faith’s PD] for age and dietary stages. To verify statistical significance, the linear mixed effect model adjusted by Bonferroni correction was performed. Only non-significant (adjusted *p*-value ≥ 0.05) pair of weeks of ages were marked on box plot. **(b)** Non-metric multidimensional scaling (NMDS) plot using the generalized UniFrac distance matrix based on relative abundance. Color-shaded ellipses indicate 95% confidence intervals for the multivariate t-distribution around the centroids of the groupings, with the number of weeks as a factor. An ellipse with a black border indicates 95% confidence intervals for each group; each group is denoted by a different shape. Group A: circle, dashed line, Group B: triangle, dash-single dotted line, Group C: square, dotted line. **(c)** Generalized UniFrac (gUniFrac) pairwise distance to the growth stage of each individual. Significance was calculated using the non-parametric Kruskal-Wallis test, adjusted by Bonferroni correction. *p*-value *< 0.05, **< 0.01, ***< 0.001.

For all four α-diversity indices, the α-diversity was lowest during lactation and increased with growth to weaning, starter, and puppy (Figure 2a). The α-diversity showed significant differences across the four growth stages in the richness and Faith’s PD indices (linear mixed-effect regression model, *p* < 0.05). For Shannon (ENS) index, no differences were observed between lactation and weaning. For Pielou’s evenness, no differences were observed between lactation and weaning and between starter and puppy. According to the ages of weeks, the upward trend of α-diversity plateaued at approximately 14 weeks of age, with a similar α-diversity level maintained thereafter.

### 3.2 Growth stage-associated changes in gut microbiota β-diversity in healthy military dogs

We found high dispersion of individuals within the same age group on the NMDS plot during the lactation-weaning period (4–6 weeks), which decreased as dogs grew (Figure 2b). At 8–11 weeks, during the starter period, the dispersion degree continuously decreased in the NMDS plot. We quantitatively measured the change in gut microbiota composition using the gUnifrac distance (Figure 2c). The dispersion of individuals was significantly smaller at 14 weeks of age compared to other ages (0.24, *p* < 0.05, *t*-test) (puppy stage). From the lactation to the weaning stage (4–6 weeks), the average gUniFrac distance was 0.56. These were significantly higher than the average values of 0.24–0.37 (minimum to maximum), found after the starter stage (8–28 weeks) (*t*-test, *p* < 0.05). Additionally, gUniFrac pairwise distance of 4–6, 6–8 weeks of ages were significantly higher compared to that of 8–11, 11–14, 14–21, and 21–28 weeks of ages. In the microbial community difference analysis, no significant change was identified from four periods; 4–6, 8–11, 11–14, 21–28 weeks of ages (PERMANOVA, *p* > 0.05) (Supplementary Table 1). Except for four periods, significant differences were identified across the ages of weeks. Although PERMANOVA results revealed significant differences between groups, Beta Dispersion analysis showed heterogeneity in within-group dispersion, which may influence the interpretation of PERMANOVA results.

### 3.3 Changes in the gut microbiota taxonomic composition during the growth of healthy military dogs: phylum- and family-level analyses

**FIGURE 3:**
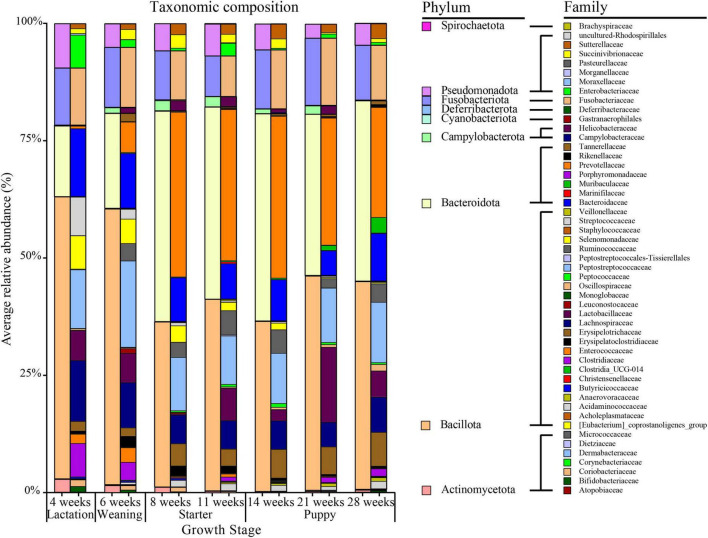
Taxonomic composition by age (in weeks) of healthy groups at the phylum and family level. On the 100% stacked bar chart for each age group, the phylum-level and family-level taxonomic compositions are given on the left and right, respectively.

The five major phyla with high average relative abundance in canine microbiota, from the lactation to the puppy stage, were Bacillota (avg. 49.89%), Bacteroidota (avg. 29.38%), Fusobacteriota (avg. 11.88%), Pseudomonadota (avg. 6.27%), and Actinomycetota (avg. 1.38%) (Figure 3 and Supplementary Figure 2). The average relative abundance of Bacillota was 60.23% during the lactation stage (4 weeks) but decreased to 42.23% at the puppy stage (14–28 weeks). The Bacteroidota accounted for 15.12% and 20.35% of the taxa during the lactation (4 weeks) and weaning (6 weeks) stages, respectively, and nearly doubled to 40% after the starter stage (11 weeks), which was sustained until the puppy stage (28 weeks, 40%). The Bacillota/Bacteroidota (B/B) ratio was 3.98 and 2.90 in the lactation and weaning stages, respectively, decreasing to 0.89 and 1.08 in the starter and puppy stages, respectively. The Actinomycetota showed average relative abundances of 2.80%, 1.55%, and 1.19% at 4, 6, and 8 weeks of age, respectively, decreasing to less than 0.5% on average at the starter stage (11 weeks). Two phyla, Fusobacteriota and Pseudomonadota, maintained a constant relative abundance regardless of the growth stage.

**FIGURE 4:**
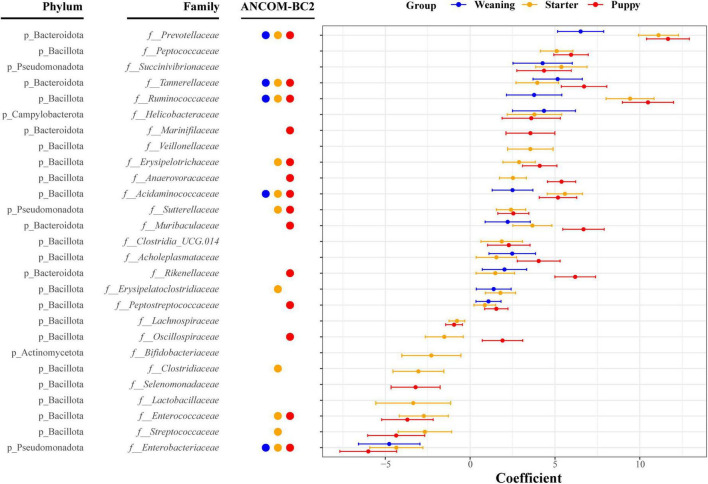
Thirty-four bacterial families significantly associated with growth stages based on absolute regression coefficients. Associations were adjusted for littermate group and sex as fixed effects, with individual sample variables as random effects. Circles and bars represent the regression coefficients and their 95% confidence intervals, respectively.

In the differential relative abundance analysis with the reference of lactation stage, we identified 27 families which are significantly associated with the growth stages (Linear regression model, *q* < 0.05) (Figure 4). Overall, weaning, starter, and puppy stages were associated with a similar set of families, with regression coefficients showing an increasing pattern as dogs grew from weaning to puppy stages. Amongst the largest positive coefficients, the coefficient of *Prevotellaceae* was 6.5 for weaning, 11.1 for starter, and 11.7 for puppy stage. The coefficient of *Ruminococcaceae* was 3.8 for weaning stage, 9.4 for starter, and 10.5 for puppy. The coefficient of *Muribaculaceae* was 2.2 for weaning, 3.7 for starter, and 6.7 for puppy stage. Amongst the largest negative coefficients, the coefficient for *Enterobacteriaceae* was −4.8 for weaning, −4.3 for starter, and −6.0 for puppy stage. The coefficient for *Enterococcaceae* was −2.3 for starter and −3.7 for puppy stage. Among 27 differently abundant families identified by MaAsLin2 analysis, the significances of 17 families remained robust in the ANCOM-BC2 analysis. Specifically, the absolute abundances of five families—*Prevotellaceae*, *Tannerellaceae*, *Ruminococcaceae*, *Acidaminococcaceae*, and *Enterobacteriaceae*— were significantly different in the weaning, starter, and puppy stages compared to the lactation stage. In contrast, the absolute abundances of 12 families, including *Enterococcaceae*, *Sutterellaceae*, and *Peptostreptococcaceae*, were significantly different in the starter and puppy stages compared to the lactation stage.

### 3.4 Changes in the gut microbiota taxonomic composition during the growth of healthy military dogs: prevalent taxa at the genus level

**FIGURE 5:**
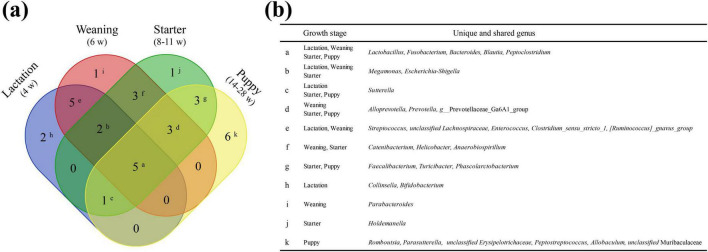
Unique or shared prevalent taxa among the four dietary stages. Lactation, weaning, starter, and puppy stages consist of 15, 19, 18, and 18 genera, respectively. **(a)** Venn diagram showing the number of unique or shared genera between the prevalent taxa at each stage. **(b)** The genus corresponding to each superscript is shown on the Venn diagram.

We annotated 114 genera from 116 healthy individuals, of which 32 were selected as prevalent taxa genera based on the relevant criteria (individual prevalence > 50%, average relative abundance > 1%). The five core genera identified from all four stages were *Fusobacterium* (avg. 11.57%), *Peptoclostridium* (avg. 11.01%), *Bacteroides* (avg. 10.03%), *Lactobacillus* (avg. 5.91%), and *Blautia* (avg. 4.37%) (Figure 5). For each growth stage (lactation, weaning, starter, and puppy stages), 15 (91.29%), 19 (86.98%), 18 (89.86%), and 18 (86.28%) prevalent taxa genera were identified.

### 3.5 Changes in lactic acid bacteria (LAB) relative abundance and diversity during growth

To analyze the changes in LAB composition in the canine gut microbiota, we evaluated the relative abundance of 13 LAB genera, namely *Lactobacillus*, *Streptococcus*, *Enterococcus*, *Lactococcus*, *Weissella*, *Leuconostoc*, *Vagococcus*, *Tetragonococcus*, *Pediococcus*, *Oenosoccus*, *Carnobacterium*, *Aerococcus*, and *Bifidobacterium*, at the different growth stages (Supplementary Figure 3). Among the 13 genera, seven genera (*Lactobacillus*, *Streptococcus*, *Enterococcus*, *Lactococcus*, *Weissella*, *Leuconostoc*, and *Bifidobacterium*) were identified in the gut microbiota of military dogs. The LAB relative abundance in the gut microbiota was the highest in the lactation stage (17.99%) with high abundance of *Streptococcus* and *Bifidobacterium*, during which the dogs were breastfed. As dogs grew, the LAB relative abundance decreased to an average value of 4.81% at the starter stage and 8.57% at the puppy stage (14–28 weeks). There was a sharp decline in the relative abundance of LAB to 1.69% at 8 weeks (initiation of starter stage) and to 2.97% at 14 weeks (initiation of puppy stage). Both decline valleys were partially recovered after stage adaptation. At the puppy stage, *Leuconostoc* were not identified. Notably, a higher relative abundance of the genera *Streptococcus*, *Enterococcus*, and *Bifidobacterium* was identified in the lactation (11.15%) and weaning (5.83%) stages than in the starter (0.95%) and puppy (0.55%) stages.

### 3.6 *Enterococcus faecalis* strain-level dynamics

Next, we conducted rep-PCR-based molecular epidemiologic analysis for 81 *E. faecalis* isolates from lactation- (*n* = 15), weaning- (*n* = 16), starter- (*n* = 28), and puppy- (*n* = 22) stage dogs. In total, 26 rep-PCR band patterns were identified and grouped into nine cluster types (Cluster I-IX, criteria: 80% band pattern similarity). In the lactation stage, five rep-PCR cluster types (Cluster I, II, IV, VII, and IX) were identified, which were not shared between groups A, B, and C (Supplementary Figure 4). Meanwhile, in the weaning/starter stage, 5–6 cluster types (Cluster I, II, VI, VII, IX and Cluster I, II, III, VII, VIII, IX) were identified, with Cluster I as the predominant type in all three groups. At the puppy stage, three cluster types (Cluster I, III, V) were identified, with Cluster I being the predominant type in both cases among the three groups.

By comparing the diversity of *E. faecalis* rep-PCR patterns for the four growth stages, we confirmed that the strain diversity [Shannon (*e^H^*) and Simpson (1/D)] of *E. faecalis* decreased with growth (Supplementary Figure 4). The *E. faecalis* strain diversity was the highest at the lactation stage (4 weeks of age, *e^H^* = 4.3038, 1/D = 4.7733) and lowest at the puppy stage (14–28 weeks of age, *e^H^* = 1.7796, 1/D = 1.4808). As the military dogs grew, the diversity changes of *E. faecalis* strains based on the rep-PCR showed decreasing pattern with the gUniFrac distances within group (Supplementary Figure 4).

### 3.7 Different relatively dominant functions of microbiota during growth: predictive functional profiling based on taxonomic composition

**FIGURE 6:**
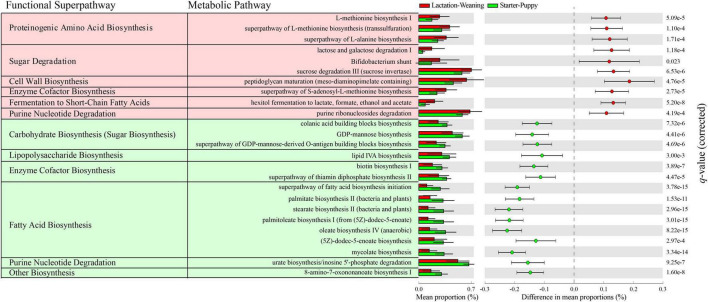
The 25 pathways with significant differences (Welch’s *t*-test with Benjamini-Hochberg FDR, *q* < 0.05) identified based on difference in mean proportions (%) > 0.1. The red- and green-shaded metabolic pathways were predicted to be relatively higher in the lactation-weaning stage group and starter-puppy stage group, respectively.

The relatively dominant functions in two groups, lactation-weaning stages and starter-puppy stages, were predicted using PICRUSt2 (Figure 6). Twenty-five pathways with significant differences (q < 0.05) were identified between the two groups. Ten of these pathways, including sugar degradation (lactose and galactose), proteinogenic amino acid (L-methionine, L-alanine) biosynthesis, and cell wall (peptidoglycan) biosynthesis, were significantly higher in the lactation-weaning group than in the starter-puppy group. The other 15 pathways, including fatty acid biosynthesis, lipopolysaccharide biosynthesis, and enzyme cofactor (vitamin) biosynthesis, were predicted to be relatively higher in the starter-puppy group than in the lactation-weaning group.

### 3.8 Gut microbiota at early life stages against CPV-2 infection: α- and β-diversity analysis

**FIGURE 7:**
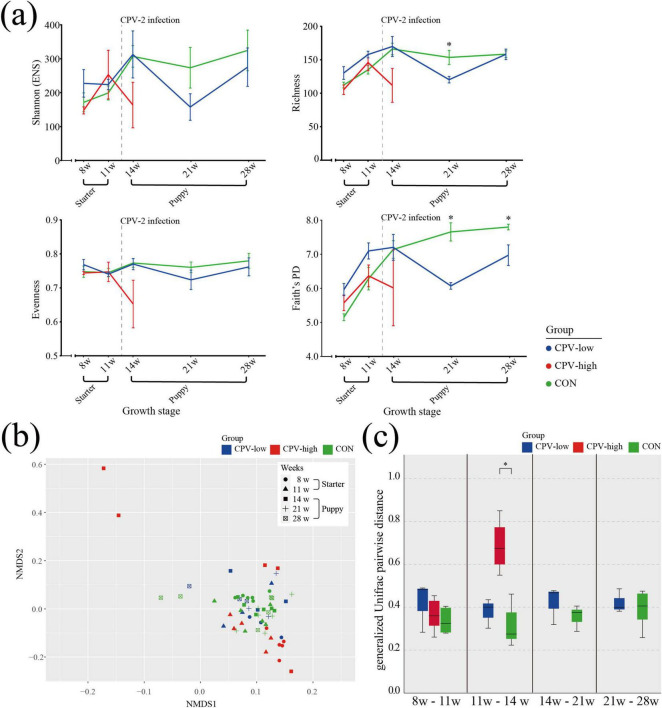
α- and β-diversity in the CPV-2 infection and healthy control dogs. **(a)** α-diversity indices, including Shannon (ENS), Richness, Pielou’s evenness, and Faith’s PD index, from 8 to 28 weeks of age. Each color represents a group; Blue: Canine parvovirus (CPV)-low, Red: CPV-high, Green: CON. Error bars show the standard error of the mean (SEM). Asterisk (*) indicates significance of differences between the control group (CON) and group CPV-low. Significance was calculated using non-parametric Kruskal-Wallis test. **(b)** Non-metric multidimensional scaling (NMDS) plot of CPV-low, CPV-high, and CON groups at 8 and 11 weeks of age before CPV-2 infection, and at 14, 21, and 28 weeks after infection. The plot was obtained based on the generalized UniFrac distance matrix. Color refers to the group (Blue: CPV-low, Red: CPV-high, Green: CON) and the shape of symbols refers to weeks of age (Circle: 8, Triangle: 11, Square: 14, Cross: 21, Squared cross mark: 28). **(c)** Pairwise distance between 11 and 14 weeks of age on the NMDS plots based on the generalized UniFrac distance matrix of the paired samples of CPV-low, CPV-high, and CON.

The gut microbiota of healthy dogs (group CON) was compared with that of dogs accidentally infected with CPV-2 at around 13 weeks of age (group CPV-low and CPV-high). At 8 and 11 weeks of age, before CPV-2 infection, α-diversity indices showed only slight differences between groups (Figure 7a). At 14 weeks of age, one week after CPV-2 infection, the α-diversity index of group CPV-high noticeably decreased compared to that of group CON, whereas that of group CPV-low was similar to that of group CON. At 21 weeks of age, 8 weeks after CPV-2 infection, the diversity of group CPV-low regarding Shannon, Faith’s PD and richness α-diversity indices decreased sharply, while that of group CON was similar to the values at 14 weeks of age, i.e., without decrease (Figure 7a). At 28 weeks of age, 15 weeks after CPV-2 infection, the α-diversity indices of individuals in group CPV-low were partially restored to a level comparable to that of group CON.

In the NMDS plot showing the β-diversity index, there were no notable differences in the distribution of individuals between the CPV-high, CPV-low, and CON groups at weeks 8 and 11 (Figures 7b, c). However, at week 14, 1 week after CPV-2 infection, a higher gUniFrac distance was observed in dogs in group CPV-high compared to that of groups CON and CPV-low. The gUniFrac distance of the paired growth ages were used to quantify the changes in β-diversity due to CPV-2 infection (Figure 7c). Except for the 11–14 w average pairwise distance (0.69 ± 0.15, 95% CI) of the group CPV-high, all pairwise distances were 0.31–0.42.

### 3.9 Recovering the potential of gut microbiota at early life stages against CPV-2 infection: taxonomic composition analysis

**FIGURE 8:**
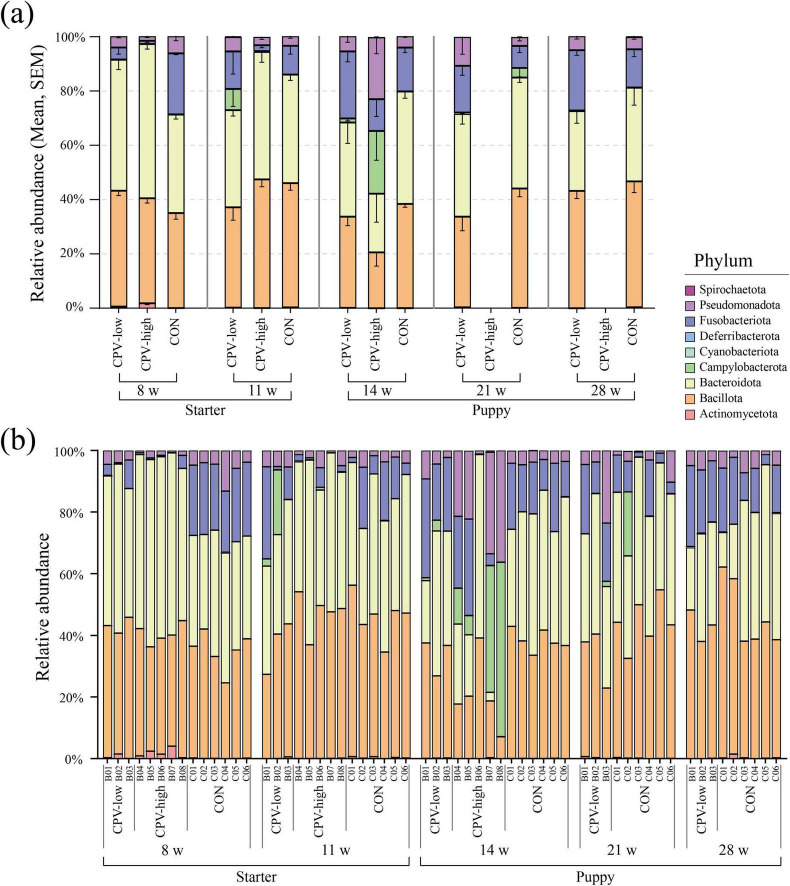
Taxonomic composition at the phylum level in Canine parvovirus-2 (CPV-2) infected and control (CON) groups. **(a)** Average relative abundance of phylum in samples used for CPV-2 infection group analysis. **(b)** Taxonomic composition for each individual sample. Each color in the bar chart represents a mean relative abundance of phylum, and the error bar represents the SEM. Only the CPV-low and CON groups were described at 21 and 28 weeks of age because all puppies in group CPV-high died before 21 weeks of age.

At 8 and 11 weeks of age, the taxonomic composition at the phylum level was similar in all the individuals from the CPV-low, CPV-high, and CON groups (Figure 8). However, at 14 weeks, after CPV-2 infection, the taxonomic composition of CPV-high was notably different from that of group CON. The taxonomic composition of CPV-low also changed compared to that of group CON but to a lesser extent than between group CPV-high and group CON.

The relative abundance of four of the major phyla in canine gut microbiota, namely Bacillota, Bacteroidota, Campylobacterota, and Pseudomonadota, was significantly changed after CPV-2 infection. The relative abundance of Campylobacterota and Pseudomonadota notably increased in the CPV-2-infected groups (avg. 0.3%–23.10%, 2.96%–22.75%), whereas that of Bacillota and Bacteroidota decreased (avg. 47.38%–20.56%, 46.98%–21.65%). According to the genus-level analysis, there was a notable inconsistency in taxonomic composition among littermates compared to that in the control group at 14 weeks of age (Supplementary Figure 5). Dysbiosis was particularly pronounced in the CPV-high group at 14 weeks old.

In the differential relative abundance analysis with the reference of CON group, we identified seven genus which are significantly associated with the CPV-high group (Linear regression model, *p* < 0.05, Table 1). No significantly differential genus was identified between CPV-low and CON groups. The relative abundance of genus *Helicobacter* was significantly higher in CPV-high group compared to CON group (regression coefficient: 8.59, linear regression model, *p* < 0.001). In contrast, the relative abundance of genus *Faecalibacterium* was significantly lower in CPV-high group than CON group (regression coefficient: −6.01, linear regression model, *p* < 0.001). The relative abundance of genus *Turicibacter* was significantly lower in CPV-high group than CON group (regression coefficient: −6.40, linear regression model, *p* < 0.001). At 21 and 28 weeks of age, the taxonomic composition of the group CPV-low was comparable to that of group CON.

**TABLE 1:** Differentially abundant bacterial genus in canine parvovirus-2 high-dose infection group (CPV-high) and control group (CON).

Bacterial genus	Abundance (%)^a^		MaAsLin2^b^		
	**CPV-high (*n* = 5)**	**CON (*n* = 6)**	**Coefficient (SE)**	***P*-value**	***q*-value**
*g__Helicobacter*	22.66	0.03	8.59 (1.93)	0.001**	0.04*
*g__Peptococcus*	0.20	0.96	−2.36 (0.51)	< 0.001***	0.04*
*g__Holdemanella*	0.21	1.66	−3.06 (0.65)	< 0.001***	0.04*
*g__Tyzzerella*	0.02	0.21	−4.33 (0.80)	< 0.001***	0.03*
*g__Erysipelatoclostridium*	0.11	0.45	−5.04 (1.11)	< 0.001***	0.04*
*g__Faecalibacterium*	0.46	7.23	−6.01 (1.16)	< 0.001***	0.03*
*g__Turicibacter*	0.06	2.07	−6.4 (1.05)	< 0.001***	0.03*

^a^Relative abundance of bacterial genus in CPV-high and CON groups.

^b^*P*-values from the generalized linear model using MaAsLin on pairwise testing between two groups. *q*-values were calculated using the Benjamini-Hochberg method for multiple testing correction.

*p < 0.05,

**p < 0.01,

***p < 0.001. SE, standard error.

### 4 Discussion

The gut microbiota is associated with the health status of the host, and microbial dysbiosis could be an important risk factor for various diseases, including metabolic and immune disorders (Carding et al., 2015; Li et al., 2017; Dogra et al., 2020). A well-established gut microbiota at the early life stages may play an important role in the host’s ability to resist disease (Kelsen and Wu, 2012). This study tracked the canine gut microbiota dynamics at early life stages against several factors, including growth, diet, external environmental exposure, and CPV-2 infection, in a birth cohort of military dogs born and raised in a controlled training environment. As the gut microbiota can be highly influenced by external factors and controlling confounding factors is essential for its thorough research (Reese and Dunn, 2018), the birth cohort study design may provide a suitable model for studying canine gut microbiota at early life stages. To our knowledge, this is the first study to analyze canine gut microbiota by 16s rRNA amplicon sequencing-based analysis with molecular epidemiology of *E. faecalis* during the early life of military dogs in a training environment.

Understanding individual taxonomic diversity and inter-individual variation could be the cornerstones for determining the stabilization status of the gut microbiota (Schmitz and Suchodolski, 2016). Here, the gut microbiota taxonomic diversity, i.e., α-diversity, increased during the lactation to starter stages and stagnated at around 14 weeks of age (puppy stage). The gut microbiota of mammals, including humans, increases in α-diversity from birth (Derrien et al., 2019). α-diversity increases plateau at certain time points during growth, and these stagnated points are used as a criterion for gut microbiome stabilization (Yassour et al., 2016). The plateaued state of individual α-diversity at the puppy stage (14 weeks) identified here implies that the individual taxonomic diversity in the gut microbiota of military dogs could be stabilized at this stage.

In the analysis of gut microbiota diversity between individuals, the highest β-diversity dispersion was observed in military dogs at the lactation and weaning stages (4–6 weeks of age). The gut microbiota of human infants is established primarily through the transmission of the mother’s gut, skin, fecal, vaginal, and breast milk microbiota (Palmer et al., 2007; Cong et al., 2016). As the naive intestinal environment of infants cannot provide adequate buffering capacity for external microorganisms, the gut microbiota received from their mothers may settle randomly and certain microorganisms may become predominant (Zakosek Pipan et al., 2020). This allows for a high diversity of microbial compositions between individuals even under the same conditions, which could be a potential cause for the high β-diversity at the lactation and weaning stages of military dogs.

During the starter stage (8–11 weeks of age), β-diversity was decreased compared to that at the lactation and weaning stages. We previously reported that a commercial diet may lower the β-diversity of canine gut microbiota (Kim et al., 2017). However, increased exposure to the external environment increases gut microbiota β-diversity (Tasnim et al., 2017). Here, meanwhile, the β-diversity of starter dogs with increased exposure to the external environment, including through play in the backyard, was lower than that of lactating and weaning dogs living exclusively indoors. Therefore, our results suggest that dietary changes may play a more significant role than housing environmental changes in expanding the gut microbiota diversity. Similarly, Carmody et al. (2015) has shown that major dietary modifications induce more pronounced shifts in gut microbiota composition in mice compared to housing environmental changes. In contrast, husbandry or environmental differences, such as variations in cage bedding or standard chow formulations, tend to produce only subtle or transient microbiota changes (Ericsson and Franklin, 2021). Taken together, dietary exert a dominant influence on gut microbiota diversity.

The β-diversity dispersion was the lowest at 14 weeks of age (puppy stage). Although the diet changed from a starter commercial diet to a puppy commercial diet between 11 and 14 weeks of age, dispersion of β-diversity decreased during this period rather than increasing. This suggests that, despite the change in diet, the overall composition of the main nutrients in the two diets remained largely similar, which may explain the observed decrease in dispersion of β-diversity at 14 weeks. Collectively, the increase in α-diversity, which plateaued at 14 weeks of age (the puppy stage), and the low β-diversity dispersion pattern at week 14 suggest that gut microbiota matures at this age and it is characterized by the homogeneity of the taxonomic composition among individuals of the same age group. Hence, the gut microbiota may have stabilized at 14 weeks, i.e., at the early life stages of military dogs. Moreover, the slight increase that followed during the puppy stage (14–28 weeks) was possibly due to increased exposure to several environmental factors with the initiation of outdoor training activities, including reconnaissance, tracking, and obstacle detection.

The *E. faecalis* genetic diversity changes obtained through the culture-based method, interestingly, showed a similar β-diversity change pattern according to the growth stages (positive correlation, Spearman’s correlation test, *P* < 0.05). Most single bacterial species constituting the gut microflora consist of a single dominant strain accounting for more than 80% of the population (Truong et al., 2017). Additionally, the strain-level diversity is strongly influenced by the host’s geographic distribution (Yan et al., 2020). Previous reports and our results suggest that the gut microbiota shares taxonomic composition between individuals and the dominant strains that occur during stabilization throughout the early life stages of dogs. Introduction and elimination of new strains or strain-level dominance investigation are extremely difficult using 16s rRNA-based taxonomic analysis. Therefore, genome-based studies supported by culture-based studies may provide a better understanding of microbiota changes.

Rep-PCR clusters of *E. faecalis* were not shared between dogs during the lactation stage. However, as dogs grew, rep-PCR clusters of *E. faecalis* were shared between dogs, which implied decreased individual gut microbiota variation. As military dogs grew, the diversity of *E. faecalis* strains decreased, and the shared strain composition between individuals increased. *E. faecalis* is a species found in all environments, including soil, water, animals, and plants (Byappanahalli et al., 2012). Therefore, the *E. faecalis* present in military dogs can spread between the groups through various environments, including the shared playground soil and training tools. Additionally, some clusters were temporarily detected only at specific growth stages and in the littermate group (e.g., cluster IV at the lactation stage in group A). The *E. faecalis* strain belonging to this cluster may have been introduced from the external environment and is a transient member of the gut microbiota.

In a comparative analysis of relative and absolute taxa abundance across different growth stages using MaAsLin2 and ANCOM-BC2, it was found that as the military dogs grew, there was a significant increase in the abundance of Bacteroidota, particularly *Prevotellaceae. Prevotellaceae* is a member of the short-chain fatty acid (SCFA) producers known for maintaining intestinal health through its anti-inflammatory effects (Bedarf et al., 2017). Additionally, the absolute abundance of *Ruminococcaceae*, another SCFA producer, significantly increased. SCFAs contribute to intestinal health by exerting anti-inflammatory effects, promoting mucus production, and maintaining intestinal barrier integrity (Silva et al., 2020). Thus, increased SCFA production indicates enhanced health and stability of the gut microbiota. Conversely, there was a significant decrease in the absolute abundance of *Enterobacteriaceae* with, a family closely associated with dysbiosis, which reduces gut microbiota diversity and stability (Baldelli et al., 2021). Although *Enterobacteriaceae* are linked to dysbiosis, they colonize the intestines early, particularly in newborns, by depleting oxygen in the intestine during the early period of high oxygen concentration (Arrieta et al., 2014). This aids in the colonization of strictly anaerobic bacteria. Therefore, the presence of high proportions of Pseudomonadota, particularly *Enterobacteriaceae*, during early growth can be assumed to be a step in developing and stabilizing a healthy gut microbiota.

The gut microbiota has a fluid structure that is constantly changing through the interactions between constituent microorganisms, some of which change in a correlated relationship (Stubbendieck et al., 2016). Understanding specific genus changes and their correlations in the gut microbiota environment facilitates the understanding of the gut microbiota function, ecology, and physiology. Lactose-degrading LAB such as *Streptococcus*, *Enterococcus, Lactobacillus* and *Bifidobacterium*, was higher in the lactation and weaning stages than in the starter and puppy stages. *Lactobacillus* are a representative LAB known as a probiotic that benefits humans and other animals (Douillard and De Vos, 2019). *Lactobacillus* may be an alternative to antibiotics and help the host acquire nutrients and stimulate the immune system against harmful microorganisms (Vieco-Saiz et al., 2019). Lactose, the main component of breast milk, is an essential nutrient for infant growth; however, its excessive accumulation in the intestine can cause osmotic diarrhea and growth retardation (Heyman, 2000). The high LAB richness and their evenness during the lactation and weaning stages appears to aid growth by breaking down and absorbing the lactose delivered from the mother’s breast milk. However, LAB evenness and relative abundance decrease as dogs grow into the starter and puppy stages. Interestingly, sharp decline valleys in LAB abundance were observed with dietary changes at 8 weeks (introducing phase of commercial starter feeding only) and 14 weeks (introducing phase of commercial puppy feeding), with partial recovery after stage adaptation.

As LAB may have a role in preventing the colonization of pathogenic and/or opportunistic bacteria in gut microbiota (Vieco-Saiz et al., 2019), the sharp declines in LAB abundance and genetic diversity at dietary transition observed here suggest a potential risk for gut microbial dysbiosis such as diarrhea. The administration of LAB helps ameliorate gut dysbiosis (Heyman, 2000). Perturbation of the gut microbiota is one of the major causes of growth retardation (Robertson et al., 2019; Vieco-Saiz et al., 2019; Ronan et al., 2021). Our results suggest that the period of growth transition in early life stages may serve as an appropriate time point to provide supplemental therapy (e.g., probiotics) for LAB perturbation control.

Most of the predictive metabolic pathways identified as being relatively high in the lactation-weaning stage group were related to peptidoglycan biosynthesis, proteinogenic amino acid biosynthesis, and sugar degradation. Peptidoglycan and proteinogenic amino acids are essential factors for the multiplication and growth of microorganisms, and sugar degradation could provide resources for microorganisms (Egan et al., 2017; Kaznadzey et al., 2017). The increasing gut microbiota diversity and abundance in canine early life stages is accompanied by microbiota proliferation, with increasing demand for essential elements, including peptidoglycan and proteins. A lack of biosynthesis of peptidoglycan, an essential component of the bacterial cell wall, can negatively affect gut microbiota maturation by weakening the bacterial cell wall and causing bacterial cell lysis (Lovering et al., 2012). The biosynthesis of proteinogenic amino acids, including L-methionine and L-alanine, provides an essential source for synthesizing proteins necessary for bacterial growth (Aliashkevich et al., 2018). In the predictive functional analysis of starter-puppy stages, the major predicted functions were the metabolism of nucleotides, unsaturated fatty acids, and vitamins. Unsaturated fatty acids, such as oleic acid, have an antibacterial action (Zheng et al., 2005), and lipid IV_*a*_ lipopolysaccharide evades the host immune system (Steimle et al., 2016). Synthesis of vitamin B, including biotin and thiamine, which are the main pathways at these stages, plays an important effect in gut microbiota composition by providing micronutrients (Yoshii et al., 2019). Our results suggest that the gut microbiota functions were mainly those contributing to increase microorganism diversity and richness and gut microbiota maturation during the lactation and weaning stages. Functions for maintaining the gut microbiota structure predominated in the starter-puppy stages, in which the gut microbiota was already stabilized. The function of maintaining the stability of gut-microbiota may also help with its resilience against microbial perturbation and dysbiosis, and further enhance host immunity through a healthy gut microbiota.

Canine parvovirus-2 infection, the most common cause of viral enteritis in dogs, occurs frequently in puppies younger than 6 months of age (Macintire and Smith-Carr, 1997; Silverstein and Hopper, 2009). The pathogenesis of CPV-2 infection mainly includes the destruction of the crypt epithelial cells of the intestine (Hall, 2012). The destruction of intestinal structures such as crypts and villi, where gut microbiota mainly colonizes, leads to significant changes in the gut microbiota composition. Here, we analyzed the recovering potential of gut microbiota by comparing the gut microbiota of a healthy group (group CON) with that of dogs accidentally infected with CPV-2 at 14 weeks of age (group CPV-low and group CPV-high). In detail, the CPV group was subdivided based on their CPV-2 infectious dose compared with 16S rRNA (Supplementary Figure 1). Additionally, the CPV-high group was excluded from the analysis at 21 and 28 weeks of age due to the death of dogs.

Our results suggested that CPV-2 infection may serve as a potential risk factor for gut microbiota dysbiosis, altering the α- and β-diversity and the taxonomic composition at the early phase of infection. Further, the degree of gut microbiota dysbiosis was correlated with the CPV-2 infection dose. Group CPV-high showed significant differences in α- and β-diversity and the taxonomic composition of gut microbiota when compared to group CON. Group CPV-low showed no significant differences in α- and β-diversity relative to group CON but there was a marked change in taxonomic composition, although to a lesser extent than that in group CPV-high. Compared to group CON, an increase in the relative abundance of Pseudomonadota was observed in the composition of gut microbiota in group CPV-high and CPV-low. Pseudomonadota include representative pathogenic genera such as *Escherichia*, *Salmonella*, and *Vibrio* (Mukhopadhya et al., 2012). *Helicobacter*, are normal indigenous gut microbiota microbes (Pickard et al., 2017). In contrast, *Faecalibacterium*, which was significantly elevated in the CON group, helps reduce intestinal inflammation and produces butyrate, contributing to maintaining a healthy gut microbiota through its probiotic activity (Leylabadlo et al., 2020). Additionally, *Turicibacter* is known to produce SCFAs that play a crucial role in maintaining the health of the intestinal mucosal cells (Yang et al., 2020). This suggests that in the CPV-high group, where the proportions of *Faecalibacterium* and *Turicibacter* are lower, the levels of SCFAs that help sustain gut health might also be reduced.

As no noticeable difference was found in the CPV-low group compared to the CON group immediately after CPV-2 infection, it is interesting that the α-diversity of group CPV-low was notably lower than that of the CON group 8 weeks after CPV-2 infection. Antibiotic use is an important risk factor for dysbiosis, and maximal dysbiosis occurs 2–3 days after antibiotic administration (Kelly et al., 2021). Here, antimicrobial treatment was provided to eight dogs in groups CPV-low and CPV-high immediately after CPV-2 infection detection (at 13 weeks and 6 days of age), and continued for approximately 2 weeks. Therefore, the diversity decreases 8 weeks after CPV-2 infection may be due to perturbation by the antimicrobial treatment applied. Antibiotic application for diarrhea treatment in ruminant delays diversity development and disrupts the stability of early-life gut microbiota (Ma et al., 2020). At 28 weeks of age, approximately 15 weeks after antibiotic administration, the gut microbiota of group CPV-low was comparable to that of group CON regarding α- and β-diversity and taxonomic composition. Intestinal infection and antibiotic application are representative examples of pulsed-perturbations of gut microbiota causing short-term changes, but a well-established and stabilized microbiota has resiliency and can return to its original state (Sommer et al., 2017). Collectively, our results suggest that the disrupted gut microbiota of CPV-low group, due to CPV-2 infection followed by antibiotic treatment, could be recovered to a relatively normal state (resilience) at week 28.

One limitation of this study was the relatively small sample size, which may limit the statistical power of the analyses. Although a linear mixed model was applied to explore the effect of age, the small sample size could reduce the robustness and generalizability of the results. To improve the statistical validity and reliability of future studies, a larger sample size is required for detecting significant effects and interactions. Another limitation was the lack of paternal lineage information, which could contribute to genetic variability. However, given that the primary focus of this study was the microbiota composition and its temporal changes, we believe that this limitation had minimal impact on the validity of our findings. Future studies could benefit from incorporating detailed genetic background data to explore potential interactions between host genetics and microbiota. Despite these limitations, this study provides a valuable foundation for understanding the growth and dietary changes in gut microbiota in dogs. By employing robust analytical methods and controlling for confounding factors such as diet and environment, our findings offer significant insights into the developmental dynamics of the canine microbiota. These insights can serve as a scientific basis for future studies aiming to comprehensively investigate microbiota-host interactions.

## 5 Conclusion

This study revealed that canine gut microbiota undergoes rapid changes in microbial composition throughout the early life stages even in the same litter. As dogs grew older, we observed an increase in the microbial ecology diversity of the gut microbiota. Stabilization was achieved at approximately 14 weeks of age, coinciding with the puppy stage and an increase in exposure to the external environment due to systemic training. This stabilization was further corroborated by changes in *E. faecalis* genetic diversity. With heightened exposure to the external environment shared among littermate groups, we observed the propagation of *E. faecalis* strains and the introduction of new strains. Additionally, the shared core microbiome found within the same litter and in different litters emphasizes the influence of shared environments in fostering similar microbiota compositions and strain sharing among military dogs.

## Data Availability

The datasets presented in this study can be found in online repositories. The names of the repository/repositories and accession number(s) can be found below: https://www.ncbi.nlm.nih.gov/bioproject/, PRJNA800584.
