# Supplementary material for: Dynamics of the canine gut microbiota of a military dog birth cohort
Source: Front Microbiol. 2025 Mar 24;16:1481567. doi: 10.3389/fmicb.2025.1481567 (PMC11973337; doi:10.3389/fmicb.2025.1481567)
Supplement: Supplementary file 1 [file Data_Sheet_1.docx]

Supplementary Material

# Supplementary Figures and Tables

## Supplementary Figures

**
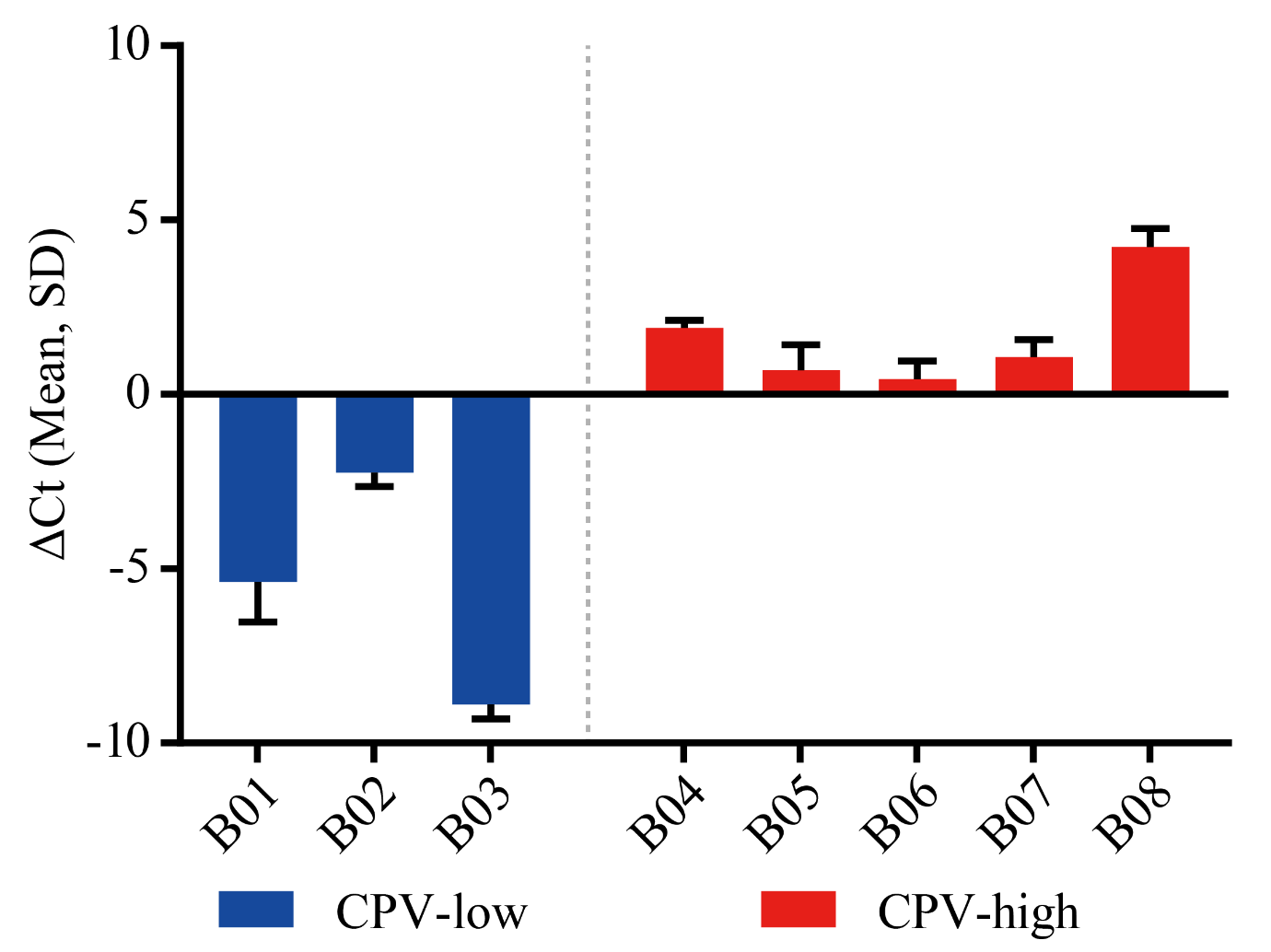
**

**Supplementary Fig. 1. Relative quantification of CPV-2.** Comparative Ct method; the amount of bacterial 16s rRNA for each individual gut microbiota was set as the endogenous control, and it was used to calculate the relative amount of CPV-2 infection.

**
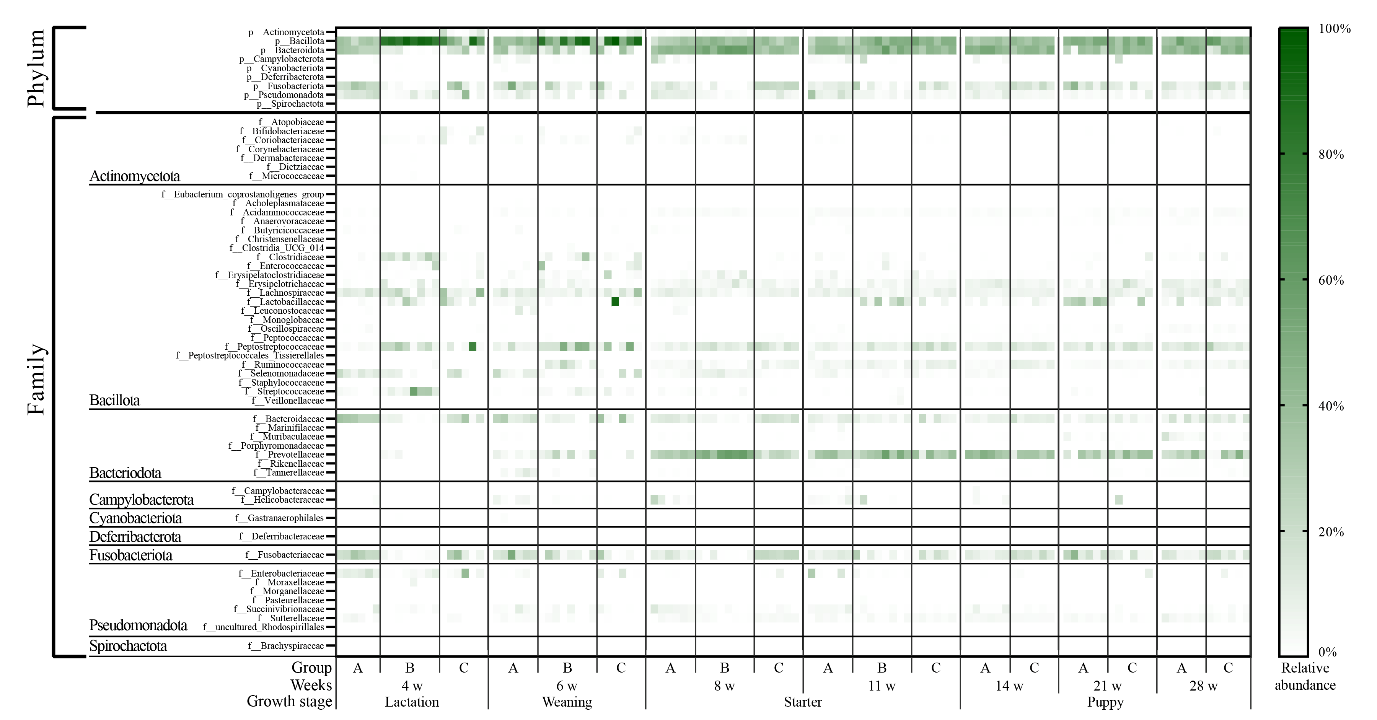
**

**Supplementary Fig. 2. Heatmap for the relative abundance at the phylum and family level of each healthy individual for a total of 116 samples**. The 4–11 weeks age group consisted of 20 puppies each, and the 14–28 age group consisted of 12 puppies each. Abbreviations: p__: phylum, f__: family

**
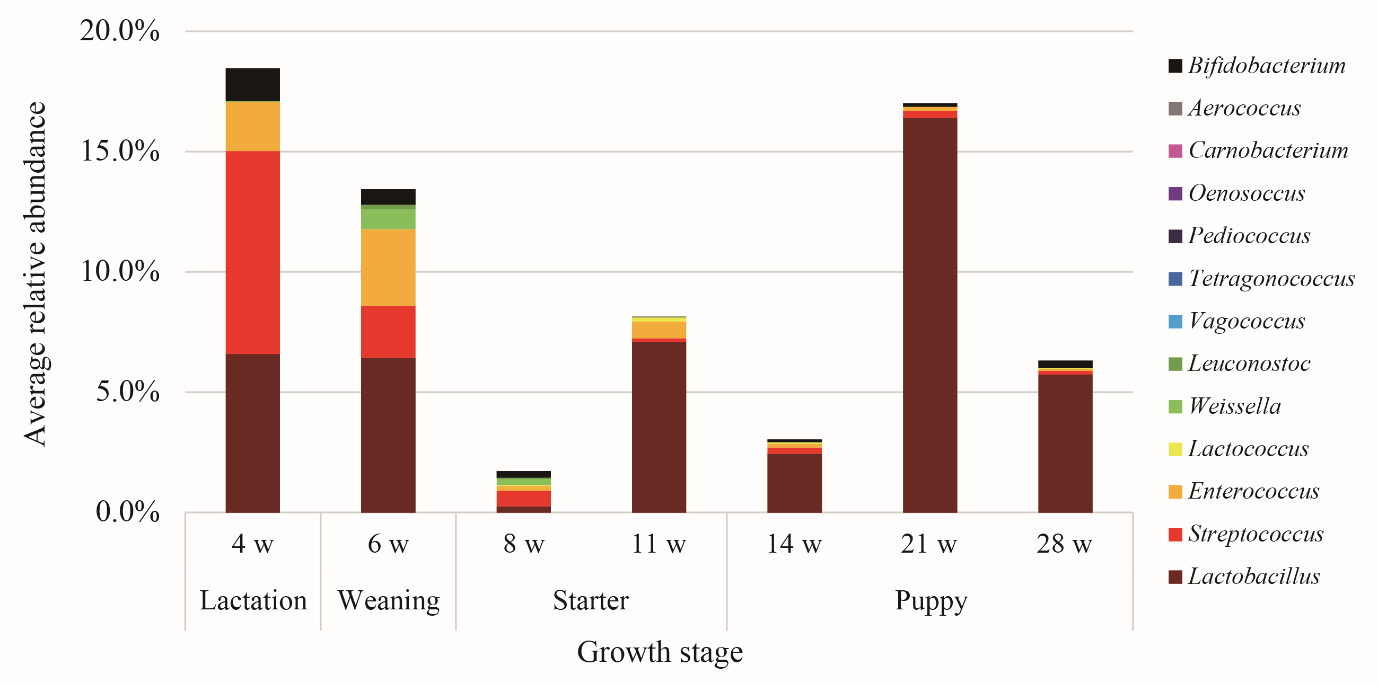
**

**Supplementary Fig. 3. Average relative abundance of 12 genera of lactic acid bacteria (genera of the Lactobacillaceae family identified in the gut microbiota) and *Bifidobacterium* in the different age groups.**

**
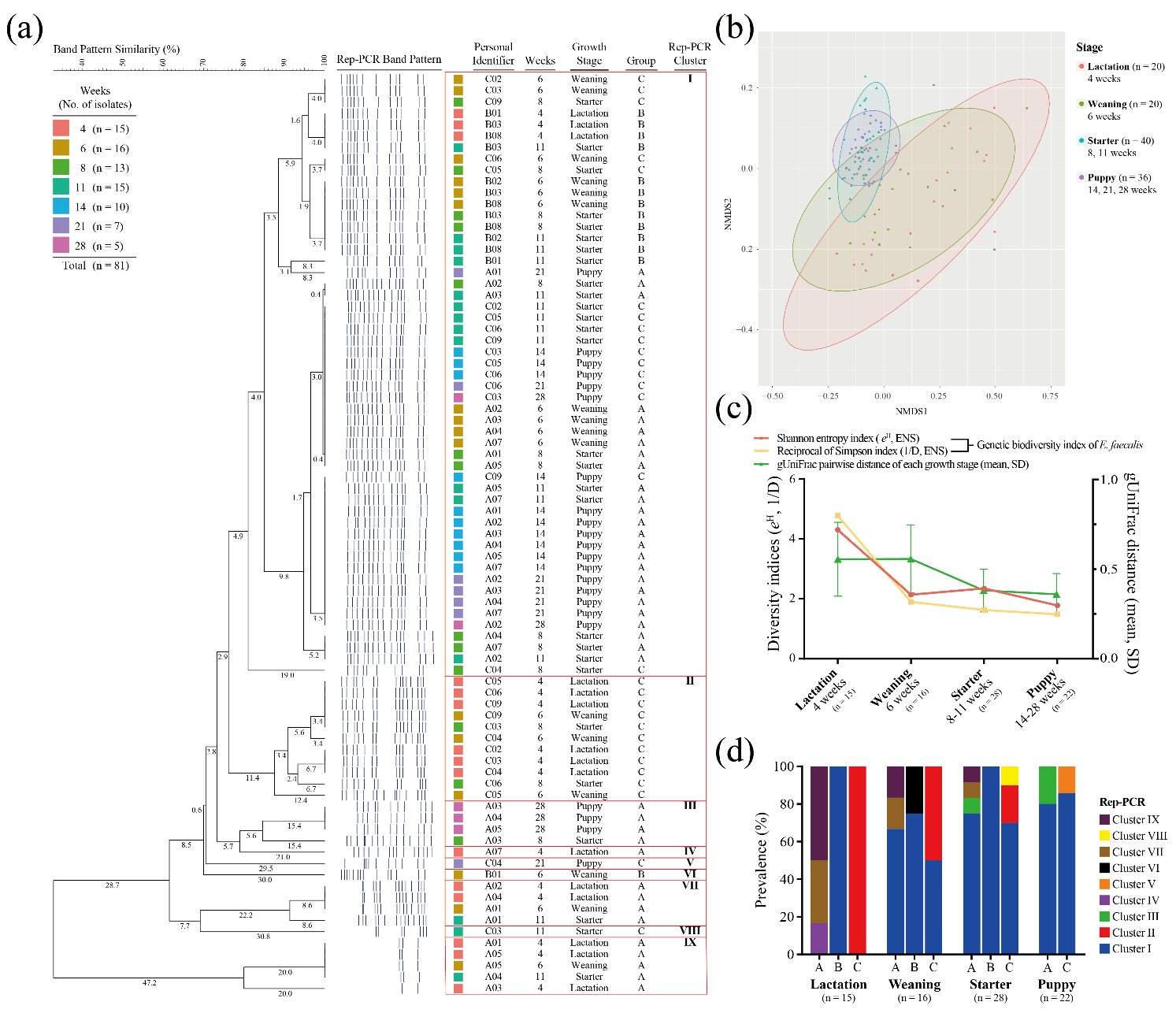
**

**Supplementary Fig. 4. Integrated analysis of genetic diversity of *E. faecalis* based on Rep-PCR with β-diversity of gut microbiota based on taxonomic composition** **of 116 healthy military dogs.** (a) UPGMA dendrogram based on Rep-PCR band pattern for *E. faecalis*. Dendrogram was generated based on 3% optimization and 1.5% tolerance based on unweighted pair group method with arithmetic mean, and clusters were generated based on 80% similarity criteria. (b) NMDS plot using the gUniFrac distance matrix. Ellipses indicate 95% confidence intervals for the multivariate t-distribution around the centroids of each stage group. (c) Line chart for genetic biodiversity indices (*e*^H^, 1/D) of *E. faecalis*, and gUniFrac distance (mean, SD). (d) Relative abundance of *E. faecalis* rep-PCR clusters for each stage and healthy group.


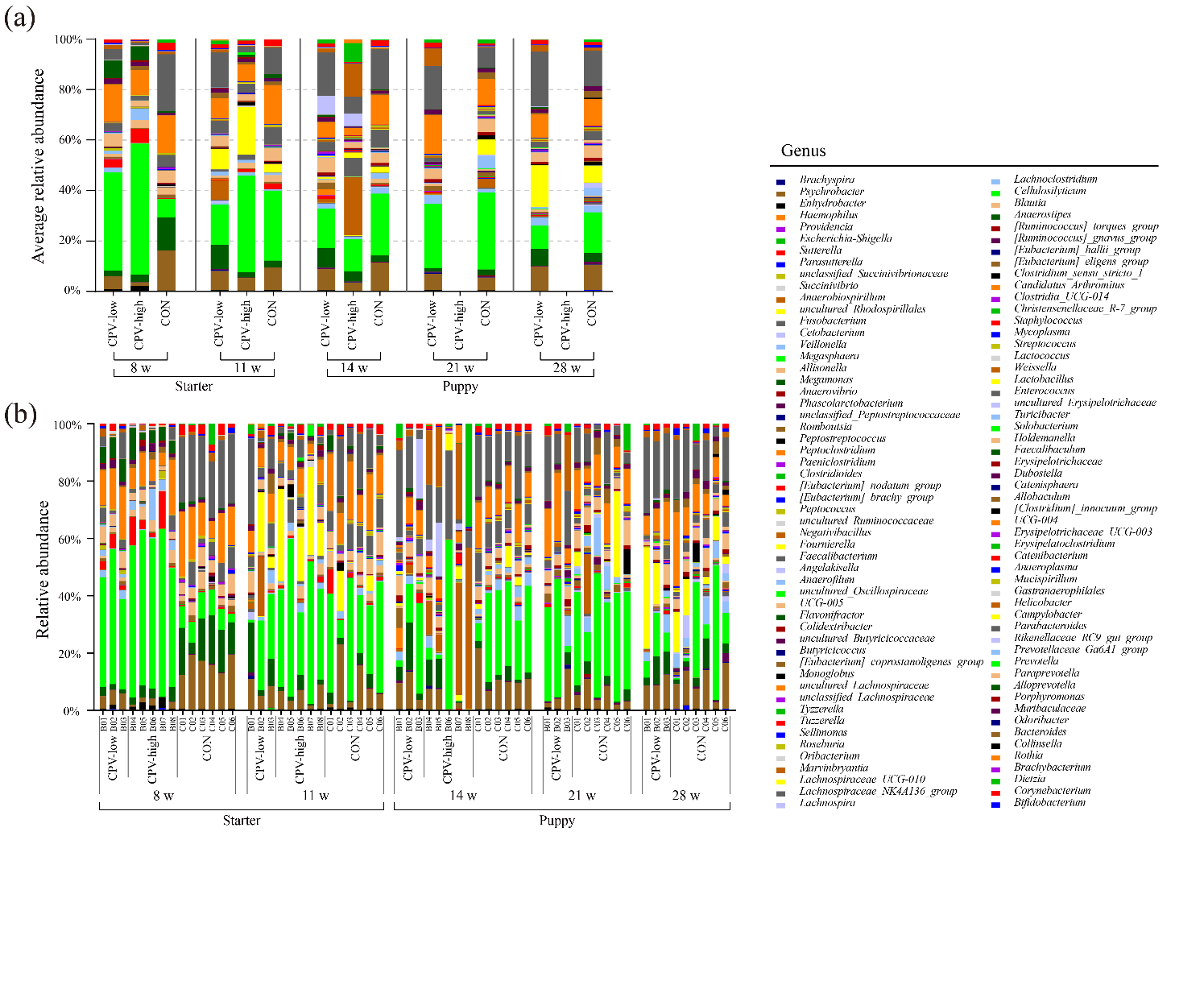


**Supplementary Fig. 5. Taxonomic composition at the genus level in CPV-2 infected (CPV-low, CPV-high) and control (CON) groups.** (a) Average relative abundance of genus in samples used for CPV-2 infection group analysis. (b) Taxonomic composition for each individual dog. Legends were listed in order from top to bottom. (e.g. *Brachyspira* was located at the top of the bar chart, and *Bifidobacterium* was located near the x-axis). Only the CPV-low and CON groups were described at 21 and 28 weeks of age because all puppies in group CPV-high died before 21 weeks of age.

**Supplementary table 1. Post-hoc multiple pairwise comparison test for PERMANOVA analysis of beta-diversity.**

| Weeks of age | |  |  |
| --- | --- | --- | --- |
| Pair 1 | Pair 2 | R^2^ | Adjusted *p*-value^†^ |
| 4 | 6 | 0.047 | 0.021^*^ |
| 4 | 8 | 0.263 | 0.021^*^ |
| 4 | 11 | 0.263 | 0.021^*^ |
| 4 | 14 | 0.302 | 0.021^*^ |
| 4 | 21 | 0.234 | 0.021^*^ |
| 4 | 28 | 0.259 | 0.567 |
| 6 | 8 | 0.171 | 0.420 |
| 6 | 11 | 0.166 | 0.021^*^ |
| 6 | 14 | 0.208 | 0.021^*^ |
| 6 | 21 | 0.165 | 0.021^*^ |
| 6 | 28 | 0.18 | 0.021^*^ |
| 8 | 11 | 0.049 | 0.021^*^ |
| 8 | 14 | 0.112 | 0.021^*^ |
| 8 | 21 | 0.196 | 0.021^*^ |
| 8 | 28 | 0.190 | 0.021^*^ |
| 11 | 14 | 0.075 | 0.021^*^ |
| 11 | 21 | 0.136 | 0.021^*^ |
| 11 | 28 | 0.145 | 0.021^*^ |
| 14 | 21 | 0.188 | 0.021^*^ |
| 14 | 28 | 0.155 | 0.672 |
| 21 | 28 | 0.086 | 0.021^*^ |

^†^ *p*-value was adjusted by using Benjamini-Hochberg method
